# Supplementary figures and images for: Evolutionary stability of topologically associating domains is associated with conserved gene regulation
Source: BMC Biol. 2018 Aug 7;16:87. doi: 10.1186/s12915-018-0556-x (PMC6091198; doi:10.1186/s12915-018-0556-x)

A

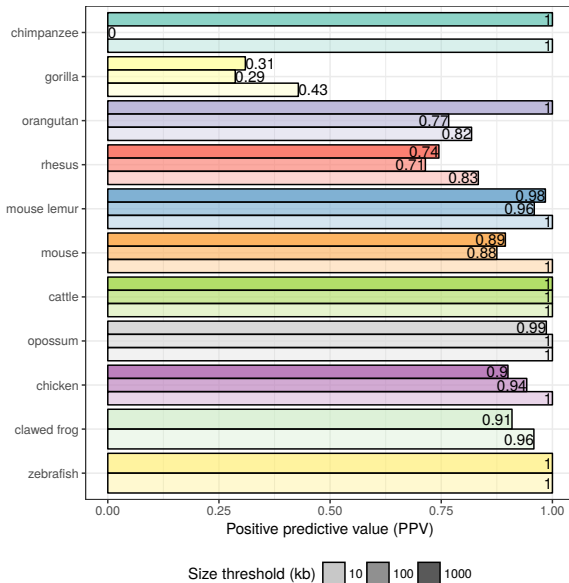

B

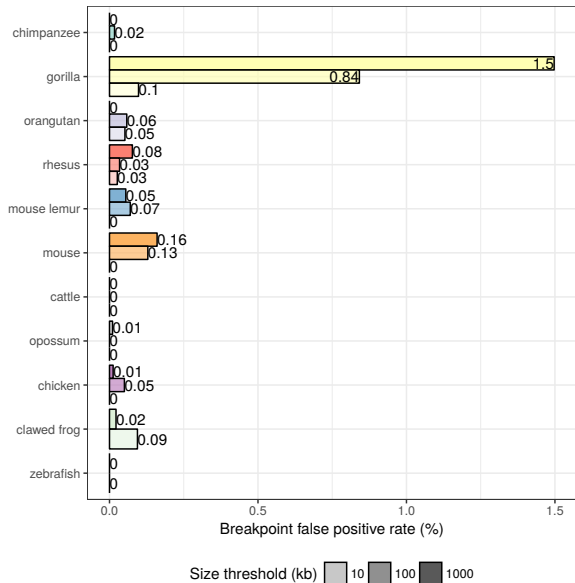

Supplement: Supplementary file 1 — Figure S1. Breakpoint identification accuracy as compared to gene synteny. Considered are adjacent pairs of human genes with one-to-one orthologs and intergenic distance below a size threshold. (A) Positive predicted value as the fraction of non-syntenic gene pairs with breakpoint from all considered gene pairs (syntenic and non-syntenic) with breakpoint. (B) False positive rate as the percent of syntenic gene pairs with breakpoint from the sum of syntenic pairs with breakpoint and non-syntenic gene pairs without breakpoint. (PDF 21 kb) [file 12915_2018_556_MOESM1_ESM.pdf]

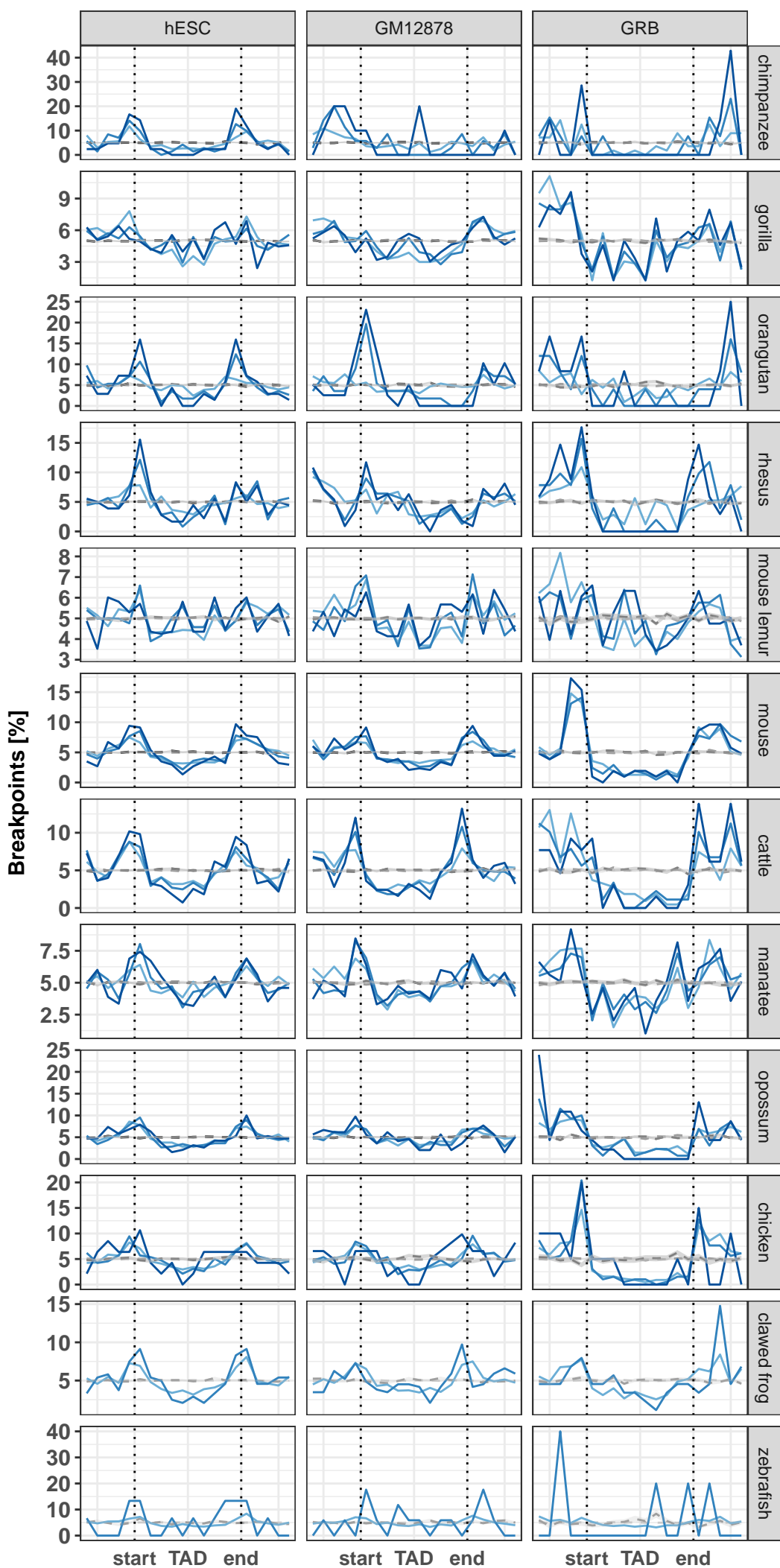

10 kb 100 kb 1000 kb

— Breakpoints - - Background

Supplement: Supplementary file 2 — Figure S2. Distribution of evolutionary rearrangement breakpoints between human and 12 vertebrate genomes around domains. Relative breakpoint numbers from human and different species (horizontal panels) around hESC TADs (left), GM12878 contact domains (center), and GRBs (left). Blue color scale represents breakpoints from different fill-size thresholds. Dotted lines in gray show simulated background controls of randomly placed breakpoints. (PDF 42 kb) [file 12915_2018_556_MOESM2_ESM.pdf]

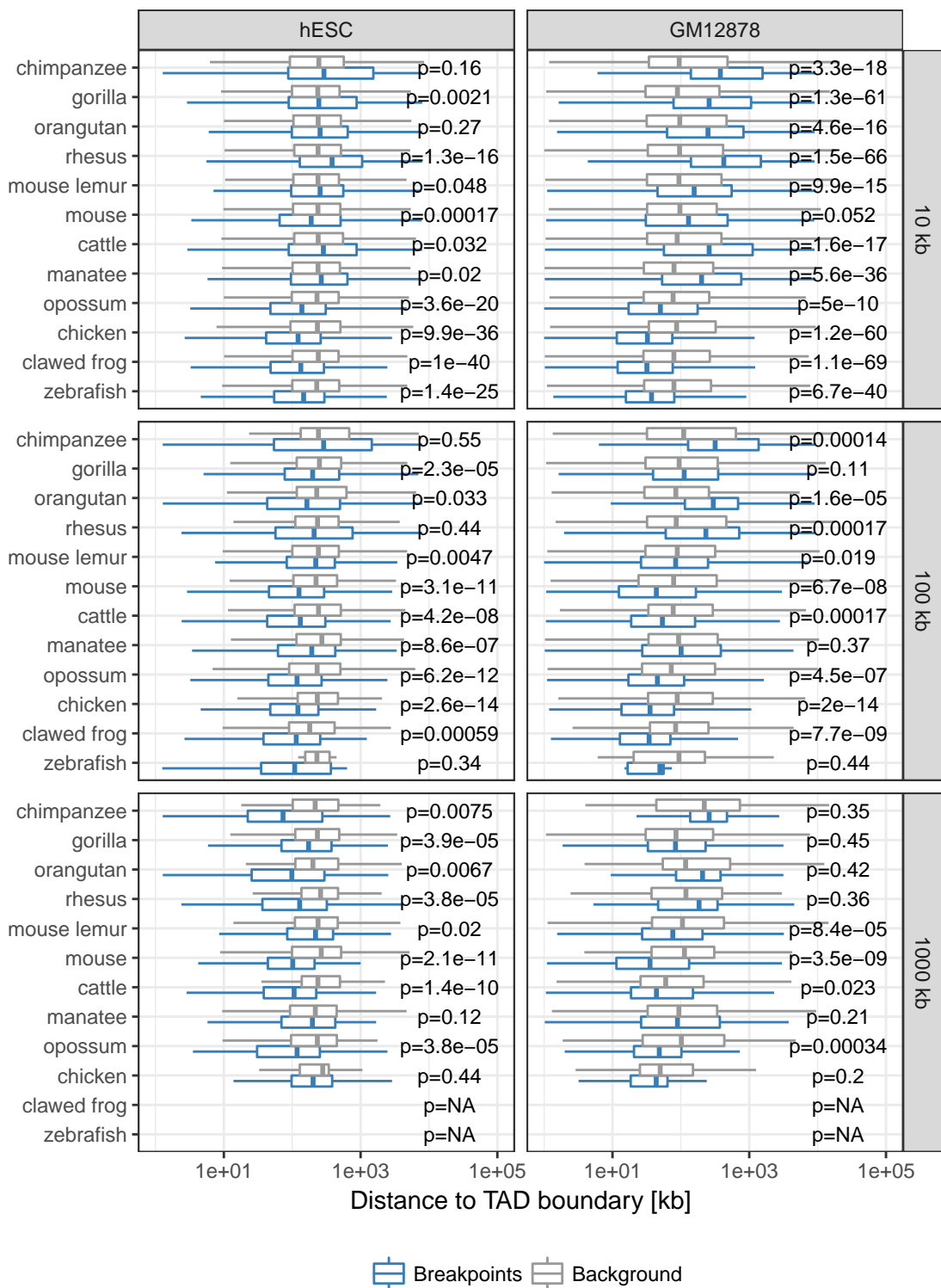

Supplement: Supplementary file 3 — Figure S3. Distance between rearrangement breakpoints and random controls to closest TAD boundary. For each species (y-axis) and fill size threshold (vertical panels) the distances from all identified rearrangement breakpoints to its closest TAD boundary (x-axis) are compared between actual rearrangements (blue) and 100 times randomized background controls (gray). The left panel shows distances to next hESC TAD boundary and the right panel distances to closest GM12878 contact domain boundary. P-values according to Wilcoxon’s rank-sum test. (PDF 14 kb) [file 12915_2018_556_MOESM3_ESM.pdf]
